# Retraction Note: Bio synthesis, comprehensive characterization, and multifaceted therapeutic applications of BSA-Resveratrol coated platinum nanoparticles

**DOI:** 10.1038/s41598-024-74394-5

**Published:** 2024-10-29

**Authors:** Shah Faisal, Muhammad Hamza Tariq, Abdullah, Sania Zafar, Zaib Un Nisa, Riaz Ullah, Anees Ur Rahman, Ahmed Bari, Khair Ullah, Rahat Ullah Khan

**Affiliations:** 1 Center for Health Research, Guangzhou Institutes of Biomedicine and Health, Chinese Academy of Sciences, Guangzhou, 510530 China; 2 Chinese Academy of Sciences, Beijing, 100049 China; 3 Institute of Biotechnology and Microbiology, Bacha Khan University, Charsadda, 24460 Pakistan; 4 Department of Biomedical Engineering and Biotechnology, Khalifa University of Science and Technology, Abu Dhabi, UAE; 5 Department of Physical Chemistry and Technology of Polymers, Silesian University of Technology, M. Strzody 9, 44-100 Gliwice, Poland; 6 Joint Doctoral School, Silesian University of Technology, Akademicka 2A, Gliwice, Poland; 7 Institute of Molecular Biology and Biotechnology, Bahauddin Zakariya University, Multan, 60000 Pakistan; 8 Department of Chemistry, Abdul Wali Khan University Mardan, Gardan Campus, Mardan, 23200 Pakistan; 9 Department of Pharmacognosy, College of Pharmacy, King Saud University, Riyadh, Saudi Arabia; 10 Department of Health and Biological Science, Abasyn University, Peshawar, 25000 Pakistan; 11 Department of Pharmaceutical Chemistry, College of Pharmacy, King Saud University, Riyadh, Saudi Arabia; 12 CAS Key Laboratory of Pathogenic Microbiology and Immunology, Institute of Microbiology, Center for Influenza Research and Early-Warning (CASCIRE), CAS-TWAS Center of Excellence for Emerging Infectious Diseases (CEEID), Chinese Academy of Sciences, Beijing, 100101 China

Retraction of: *Scientific Reports* 10.1038/s41598-024-57787-4, published online 03 April 2024

Editors have retracted this article

After publication of this article, concerns about the ethics oversight for this research were brought to the attention of the Editors. The Authors were asked to provide appropriate documentation of approval from an ethics committee for the use of samples obtained from human research participants, but were not able to do so.

Riaz Ullah disagrees with this retraction. The remaining authors did not explicitly state whether they agree or disagree with this retraction.

